# Editorial: Advances in vigilance research: exploring novel theoretical models and analytical approaches on the analysis of the vigilance decrement

**DOI:** 10.3389/fcogn.2026.1948924

**Published:** 2026-09-10

**Authors:** Juan Lupiáñez, Fernando Gabriel Luna, Daniel Smilek

**Affiliations:** 1 Mind, Brain, and Behavior Research Center (CIMCYC), Department of Experimental Psycholoty, University of Granada, Granada, Spain; 2 Universidad Nacional de Córdoba, Facultad de Psicología, Córdoba, Argentina; 3 Department of Psychology, University of Waterloo, Waterloo, ON, Canada

**Keywords:** arousal, attention lapses, cognitive control, inattention, mind wandering, motivation and resources, sustained attention, vigilance

The ability to maintain attention over prolonged periods is essential for safe and effective performance across a wide variety of tasks in modern society. Whether navigating a vehicle through heavy traffic, monitoring automated transit networks, supervising industrial systems, interpreting complex medical images, or sustaining focus within digital educational environments, humans are increasingly required to detect, discriminate, and respond to critical events over extended intervals. Yet, one of the most robust findings in cognitive psychology is that performance typically deteriorates with time-on-task, a phenomenon known as the vigilance decrement. Despite more than 75 years of research, explaining why the vigilance decrement occurs remains one of the central challenges of cognitive neuroscience and ergonomics.

This Research Topic, *Advances in vigilance research: exploring novel theoretical models and analytical approaches on the analysis of the vigilance decrement*, was motivated by the growing recognition that no single theoretical framework proposed so far fully accounts for the complex reality of the vigilance decrement. Contemporary research is increasingly characterized by a productive debate among competing and complementary explanations, including resource-depletion accounts, resource-control models, opportunity-cost frameworks, motivational approaches, and theories emphasizing mind wandering and attentional disengagement. At the same time, methodological advances are transforming how vigilance is measured, modeled, and understood. For instance, there has been a recent shift from using classic signal detection theory metrics toward psychometric curves as indices of vigilance loss. The articles collected in this Topic demonstrate that progress in vigilance research increasingly depends on integrating theoretical innovation with novel analytical and methodological approaches. The contributions in this Research Topic reflect three major themes.

A first theme emerging from the Research Topic concerns the continuing effort to clarify the *nature of vigilance* itself. The term “vigilance” is often used interchangeably with sustained attention, alertness, and arousal, sometimes to refer to the same cognitive process and other times to describe different capacities or behaviors. In their comprehensive review, Hemmerich et al. revisit these conceptual boundaries and provide an integrative synthesis of the theoretical, contextual, and neural foundations of vigilance. Their analysis complements the historical perspective provided by Klein and Feltmate, who examine how explanations of vigilance decrement have evolved since Mackworth's seminal studies across the last 75 years. Together, these contributions remind us that theoretical progress depends not only on new data but also on the development of conceptual clarity regarding the cognitive processes under investigation.

The second major theme concerns the ongoing debate about the *mechanisms underlying vigilance decrement*. Traditional resource theories attribute performance decline over time to the depletion of limited attentional resources. In contrast, newer frameworks reject simple depletion: resource-control models argue that a progressive failure of executive control allows attention to drift toward task-unrelated thoughts, while opportunity-cost and motivational theories view the decrement as a cost-benefit decision, where individuals continually evaluate the value of task engagement against alternative mental activities.

Providing evidence that bears directly on this debate, the studies by Drody et al. and Schwartzman et al. show that, in sustained attention tasks, increasing motivation and interest attenuate time-related increases in media multitasking and mind wandering, while also mitigating time-related decreases in performance. Rather than reflecting depleted cognitive capacity, these findings underscore the role of motivational factors and attentional priorities in sustaining prolonged task engagement.

Closely intertwined with motivational explanations is the increasingly influential role of mind wandering research in vigilance theory. Schwartzman et al. provide direct evidence that performance decline is accompanied by more frequent task-unrelated thoughts, even during short intervals. Extending this work into more ecological contexts, Zheng et al. identify distinct EEG synchronization signatures that differentiate on-task states from mind wandering during continuous performance. Together, these studies highlight the growing convergence between mind-wandering and vigilance research, shifting the focus from static resource availability to dynamic fluctuations in attentional engagement and resource allocation. Importantly, future research should expand explanations of vigilance failures to include an understanding of why the mind disengages from the task at hand. Task-unrelated thought may not simply impose performance costs: it may also serve adaptive functions, including memory consolidation (Simor et al., 2025; Wamsley et al., 2023) and creative thinking (Orwig et al., 2023). Understanding both the costs and benefits of mind wandering may therefore be essential for explaining why the vigilance decrement is so pervasive in everyday life and so hard to avoid.

More broadly, the emphasis on the dynamic nature of attentional states highlights the need for theoretical frameworks that characterize how attention fluctuates and adapts over time. In this regard, the Research Topic demonstrates that theoretical innovation in vigilance research extends beyond the dominant resource vs. motivation debate. In a provocative theoretical contribution, Jagacinski proposes an Adjustable Wave Model in which attentional deployment is conceptualized as a spatio-temporal sampling process operating at adjustable frequencies.

A third major theme concerns *methodological innovation*. Many traditional vigilance paradigms rely on overall detection rates or aggregate performance measures that provide a general measure of attention span (Simon et al., 2023), but limited insight into the mechanisms responsible for performance decline. As several contributions argue, advancing theory requires more precise analytical tools. Skinner and Giesbrecht advocate broader application of signal detection theory to distinguish declines in perceptual sensitivity from shifts in decision criterion. Their proposal resonates with issues highlighted by Klein and Feltmate, who revisit longstanding debates regarding the relative contributions of sensitivity and response bias to vigilance decrement. Together, these articles underscore the importance of moving beyond simple detection rates toward richer analyses of performance. Future research could further benefit from approaches such as psychometric curve modeling of executive vigilance (McCarley et al., 2026; Roman-Caballero et al., 2023) and ex-Gaussian modeling of reaction-time distributions (Román-Caballero et al., 2026).

This methodological shift is also reflected in dynamic and predictive approaches. Shelat et al. show how response-time patterns can identify impending attentional lapses and episodes of mind wandering, and EEG-based decoding methods such as those employed by Zheng et al. demonstrate the feasibility of tracking attentional states in real time. Such approaches hold considerable promise for both theoretical investigation and practical intervention, particularly in safety-critical settings.

Several contributions further illustrate the growing integration of computational modeling and neuroscience into vigilance research. Curley et al. combine electrophysiological measures with computational simulations to investigate interactions among working memory, cognitive control, and performance monitoring, while Matthews et al. highlight challenges in measuring temporal changes in attentional networks at the individual level. Together, these studies denote a broader shift toward multilevel accounts linking behavior, neural activity, computational mechanisms, and individual differences.

Taken together, the contributions to this Research Topic portray a field undergoing both conceptual refinement and methodological transformation. Rather than supporting a single explanatory framework, the Research Topic suggests that vigilance decrements arise from the interplay of attentional control, motivational dynamics, opportunity-cost evaluations, mind wandering, environmental influences, and neurocognitive constraints. Future theoretical progress will likely depend less on identifying a single dominant explanation than on developing integrative models that capture how these factors interact across tasks, individuals, and contexts.

We hope that this Research Topic stimulates further dialogue among competing theoretical perspectives while encouraging the development of increasingly sensitive behavioral, computational, and neurophysiological methods. By bringing together historical analyses, conceptual reviews, empirical studies, theoretical proposals, and methodological innovations, this Research Topic highlights the vitality of contemporary vigilance research and points toward a more integrated understanding of one of the most enduring challenges in cognitive science: explaining both the maintenance of task engagement and the adaptive dynamics of attentional disengagement.
